# Fostering Digital Life Skills Through Social Media With Adolescents in 6 German States: Protocol for an Accessibility Study According to the RE-AIM Framework

**DOI:** 10.2196/51085

**Published:** 2024-04-17

**Authors:** Elizabeth Zimmermann, Samuel Tomczyk

**Affiliations:** 1 Institute for Health Psychology Department of Psychology University of Greifswald Greifswald Germany

**Keywords:** adolescents, social media, prevention, life skills, RE-AIM-framework, mixed-methods, digital life, accessibility, innovative, utilization, teaching skill, empower, digital skill, life skill, German, digitalized, adolescent, adolescent health, study protocol, validity, innovation, leduin-program

## Abstract

**Background:**

Social media is essential in the lives of adolescents, with 97% of US teenagers engaging daily. While it facilitates communication, learning, and identity development, it also poses risks like harmful content exposure and psychological distress, particularly for adolescents in their critical developmental stage. Teaching digital life skills innovatively counters these risks, adapting traditional competencies such as decision-making, problem-solving, creative and critical thinking, communication, interpersonal skills, self-awareness, empathy, and emotional and stress management to digital challenges.

**Objective:**

This study evaluates the accessibility of the “leduin” program, a novel intervention designed to impart digital life skills through Instagram. The program aims to leverage social media’s educational potential, focusing on effective strategies to engage adolescents. Emphasizing accessibility is crucial, as it determines the program’s overall impact.

**Methods:**

The leduin program, developed through intervention mapping, applies behavior change techniques via social media for 9th and 10th graders. It is a 14-week spaced learning curriculum with daily sessions <5 minutes. Emphasizing the “reach” aspect of the reach, effectiveness, adoption, implementation, and maintenance (RE-AIM) model, the recruitment targets diverse educational settings across 6 German states, aiming for inclusivity. Recruitment will involve schools, youth centers, and therapeutic facilities. The study seeks at least 128 participants, a calculated minimum to detect medium-sized effects in the quasi-experimental design and explore varying engagement levels and program responses. Data collection includes preintervention, postintervention, and 6-month follow-up surveys, using multilevel regression, latent growth models, and qualitative analysis to extensively assess reach and gain first insights on effectiveness, acceptance, implementation, and maintenance. The study aims to reveal key factors influencing program participation and interaction; a detailed analysis of engagement patterns will reveal the effectiveness of the recruitment strategies and barriers to participation. Additionally, initial indications of the program’s impact on life skills, social media–related skills, health status, risk behaviors, and academic performance will be analyzed.

**Results:**

Recruitment was planned from May 2023 until the beginning of the leduin program in October 2023. As of March 2024, we have recruited 283 participants.

**Conclusions:**

The leduin program stands as an innovative and essential initiative in adolescent health promotion, harnessing the power of social media to teach important digital life skills. This study highlights the critical role of accessibility in the success of social media interventions. Effective adolescent engagement strategies are imperative, as they dictate the overall impact of such interventions. The insights gained from this study will be instrumental in shaping future programs, laying groundwork for a subsequent, more comprehensive cluster-randomized controlled trial. The study’s design acknowledges the limitations of the current quasi-experimental approach, including the anticipated sample size and the absence of a control group, and aims to provide a foundational understanding for future research in this field.

**Trial Registration:**

Deutsches Register Klinischer Studien DRKS00032308; https://drks.de/search/de/trial/DRKS00032308

**International Registered Report Identifier (IRRID):**

PRR1-10.2196/51085

## Introduction

### Background

Social media, a staple in the lives of 97% of US teenagers [1], facilitates communication, learning, and identity development for adolescents [2-5]. However, the functions and the lack of censorship on these platforms can lead to risks such as addiction, psychological distress, and exposure to harmful content [6-8]. Given the ongoing developmental stage of adolescents [9], they frequently lack the essential social-emotional and cognitive skills required for functional and constructive engagement with social media [10]. These challenges underscore the importance of building social media literacy, a crucial skill that enables adolescents to navigate digital content critically and responsibly [11]. Enhancing media literacy involves teaching adolescents to understand and manage the psychological impacts of digital interactions, including the development of self-regulation and critical thinking skills [12-14].

The widespread use of social media also offers a unique opportunity for preventive health interventions. As a cost-effective platform with a broad reach [3,15-17], social media can play a pivotal role in promoting mental and physical health [18-22]. Its accessibility makes it particularly valuable for reaching socially disadvantaged adolescents, who may engage more frequently with digital platforms, often in dysfunctional ways [23-26]. Thus, social media emerges as a critical tool in enhancing equity in health promotion.

While recognizing the potential of social media in adolescent health promotion, the limited evidence base highlights the need for careful consideration of practical, methodological, and ethical implications [22,27-30]. Current studies focus on engaging content and tailored interventions for diverse needs [20,29,31,32] but often lack a comprehensive, evidence-based approach that integrates psychological mechanisms and behavioral change techniques [30]. This gap extends to the challenge of effectively recruiting adolescents, especially for programs addressing sensitive health issues [33,34]. Recruitment complexities arise from stigma and the need to align with adolescents’ varied interests and obtain parental consent [35-37]. Addressing these challenges necessitates multifaceted strategies: empathetic engagement; using diverse channels; and clearly communicating the benefits while ensuring trust, confidentiality, and cultural sensitivity [38,39]. These methods emphasize the need for a supportive and safe environment, messages that resonate with adolescents, and the involvement of the broader community in the recruitment process for prevention programs.

### Digital Life Skills in the Age of Social Media

Modern prevention strategies must encompass the growing convergence of analog and digital life, acknowledging the diverse challenges in both realms. In this context, promoting life skills emerges as a valuable and effective approach, contributing significantly to overall well-being, academic success, and the reduction of risk behaviors and chronic diseases [40-44].

Life skills, as defined by the World Health Organization [45], include decision-making, problem-solving, creative and critical thinking, communication and interpersonal skills, self-awareness, empathy, and managing emotions and stress. Traditional programs, despite their benefits, face challenges such as high costs and limited accessibility [46,47]. The digital era offers new avenues through e-learning and apps to effectively impart these skills while addressing issues such as substance abuse [48-53] but also necessitates developing digital skills to tackle digital challenges [11,12,54].

### Development of a Digital Life Skills Program (Leduin)

#### Overview

Consequently, we have developed the “leduin” program, using the social media platform Instagram to teach digital life skills in a low-threshold and interactive manner, seamlessly integrated into everyday life. In doing so, we followed the intervention mapping methodology [55], which directs the conceptualization, development, and implementation of health promotion programs. The leduin program is a digital intervention specifically designed to empower adolescents in the 9th and 10th grades with crucial digital life skills to navigate challenges presented by social media and everyday life. The following steps were undertaken to develop the leduin program (E Zimmermann and S Tomczyk, unpublished data, 2024).

#### Logic Model Development

The development of the leduin program began with a comprehensive logic model, addressing the complexities of adolescent life in the context of social media. This model focuses on developing digital life skills that enhance health, reduce risk behaviors, and improve school performance, particularly targeting 9th and 10th graders. This timing is strategic as life skills are especially beneficial and more effectively imparted during such transitional periods in a student’s life [56]. The model was informed by focus groups with 67 adolescents aged 14 to 17 years and heterogeneous educational backgrounds.

#### Psychological Mechanisms Identification

The second step involved identifying psychological mechanisms vital for behavioral change and digital life skills development. The leduin program integrates (1) personal variables such as attitudes and self-efficacy, based on social cognitive theory and the theory of planned behavior [57-59]; (2) environmental factors including social norms and support [60,61]; and (3) information processing mechanisms to optimize message impact [62]. Additionally, it focuses on (4) activating resources to support the development of digital life skills [63].

#### Developing Methodological Framework and Intervention Design

For the third step, we crafted a methodological framework using the behavior change taxonomy [64], tailored to social media context. Influenced by research on social media [65], behavior change techniques in health programs [66], and user preferences [67], we chose Instagram for its interactivity and popularity [3,68]. The program integrates behavior change techniques within Instagram, encompassing (1) content sharing through posts and stories for information dissemination, (2) interactive features such as quizzes for behavioral regulation, (3) material and social incentives to enhance engagement, and (4) social processes such as polling and commenting to encourage participation. These components collectively support habit formation, problem-solving, and identity development related to behavior change (see Table 1 for detailed mechanisms and associated features), with the selection process also being guided by adolescent focus group feedback.

**Table 1:** Mechanisms, exemplary behavior change techniques, and associated exemplary social media features within the social media–based life skills program (leduin).

Mechanism	Exemplary behavior change techniques	Exemplary social media features
Content sharing	Information about (1) health consequences (5.1) Information about (2) social and environmental consequences (5.3) Information about (3) emotional consequences (5.6) Pros and cons (9.2) Salience of consequences (5.2) Instruction on how to perform the behavior (4.1) Verbal persuasion about capability (15.1) Credible source (9.1)	Content sharing methods: posts, tweets, and stories using text, image, and video
Behavioral regulation	Self-monitoring of behavior (2.3) Self-monitoring of outcomes of behavior (2.4) Discrepancy between current behavior and goal (1.6)	Interactive elements like quizzes, sliders (similar to Likert-scale), templates, and question stickers
Incentives	Material incentive (behavior; 10.1) Social reward (10.4)	Raffles and prizes Likes, comments, and direct messages
Social processes	Social comparison (6.2) Social support (unspecified; 3.1) Restructuring the social environment (12.2)	Sliders, polls, and direct messages Direct messages, likes, and group chats Community and account recommendations
Processes initiated	Habit formation (8.3) Problem-solving (1.2) Goal setting (behavior; 1.1) Identity associated with changed behavior (4.1)	Push notifications

#### Program and Module Structuring

In the fourth step of the intervention mapping approach, the leduin program’s structure and modules were developed. It resulted in a 14-week program that encompasses modules on individual, social, and health skills, detailed in Textbox 1. Each module introduces, specifies, applies concepts to risk situations and relates them to everyday life, aligning with World Health Organization life skills education guidelines [69]. Modules cover areas such as emotional and stress management, communication skills, and digital safety. Content is delivered through Instagram stories and feeds using short videos, images, quizzes, and surveys to foster active learning. Additionally, the program incorporates weekly self-care and stress management activities and interactive challenges with prizes to boost engagement. An accompanying analog workbook with journaling activities complements the digital content. This dual approach ensures comprehensive life skills training, emphasizing concise, daily content to promote spaced learning [70], contrasting with traditional massed learning methods.

**Contents of the 14 modules of the 14-week social media–based life skills program (leduin) divided into individual, social, and health skills.**

**Individual skills (modules 1-4):**

- Strengths, goals, emotions, and stress

**Social skills (modules 5-8):**

- Communication, needs, boundaries, identity, and peer pressure

**Health skills (modules 9-12):**

- Risks, addictions, digital violence, and information

**Cross-module:**

- Self-care, stress reduction, problem-solving, and self-reflection
- Modules 0 and 13 contain introduction and conclusion

### Pilot Study of the Leduin Program

In a pilot study, we assessed the feasibility, acceptance, and engagement of the leduin program, implementing it with 101 students of 9th and 10th-grade in high schools and secondary schools in North Rhine-Westphalia and Lower Saxony, Germany, from November 2022 to February 2023 (E Zimmermann and S Tomczyk, unpublished data, 2024). A total of 13 interviews with participating adolescents aged 14 to 17 years and 6 interviews with their teachers were conducted to evaluate acceptance and initial effectiveness. These qualitative findings indicate that the social media–based prevention program was successfully conducted, well-received by participants, and executed with high commitment. Participants reported positive subjective learning outcomes in social media use, self-care, and stress reduction and perceived the leduin program as personally beneficial and enriching. Although the program yielded favorable results, its accessibility presented a substantial hurdle in the recruitment phase. We conducted informational sessions in schools and a youth center to educate adolescents about the program, enabling them to make informed choices about participating in their leisure time. However, the participation rates varied significantly among schools, with certain classes showing high engagement, while others had minimal involvement. This variation underscores the importance of developing effective strategies to engage adolescents in social media–based prevention programs. Consequently, in the fifth step of the intervention mapping approach as the subject of this study, our focus is to examine and enhance accessibility to guarantee effective program execution.

## Methods

### Overview

The objectives of the accessibility study for the leduin program, grounded in the reach, effectiveness, adoption, implementation, and maintenance (RE-AIM) framework [71], primarily focus on evaluating the first dimension of the framework—“reach”—to identify effective strategies for engaging adolescents in digital life skills interventions on social media. This includes reaching a diverse demographic of 9th and 10th graders, encouraging participation, and ensuring sustained engagement. While also considering the remaining RE-AIM aspects—effectiveness, adoption, implementation, and maintenance—the study mainly seeks to understand the characteristics and engagement levels of participating adolescents and to identify what drives active or low engagement. This understanding is critical for refining recruitment strategies and enhancing the impact of social media–based health interventions for youth.

### Reach

The focal point of our study is to encompass a broad and representative demographic of 9th- and 10th-grade adolescents. To achieve this, we are targeting a diverse population from various school types, including grammar schools, comprehensive schools, secondary schools, and independent schools. Moreover, recognizing the importance of reaching adolescents undergoing high stress, recruitment efforts are also extended to youth centers and therapeutic facilities. We will explore the characteristics of adolescents who have consented to participate. Based on insights from the pilot study, we anticipate varying levels of engagement among the participants: some may actively engage with the program’s social media content, while others may have minimal to no interaction although initially interested. This variance allows for a quasi-experimental design, using engagement levels (ranging from none to high) as the basis for group assignment. Such a design is capable of highlighting differences between these engagement groups, and we will explore the predictors that are associated with active engagement or no or low engagement. We will also conduct a qualitative process evaluation to explore the facilitators and barriers encountered during recruitment. This will involve the recruitment team documenting feedback about the program and information event, as well as the reasons participants choose to join or decline participation. This documentation will be captured both in written form and verbally.

### Effectiveness

The program’s effectiveness will be evaluated by its impact on life skills, social media–related skills, health status, risk behaviors, and school performance. Using the quasi-experimental design, we aim to test the hypothesis that enhancing digital life skills through the intervention leads to improved social media skills and reduced risk behaviors. To detect medium-sized effects, a power analysis indicates the need for 128 adolescent participants (power=0.8, α=.05), aligning with existing evidence of medium effect sizes in digital interventions (d=0.30 to 0.76) [72]. This evaluation will provide crucial data for potential effectiveness, shaping the direction of future trials.

### Adoption

The study will evaluate the proportion of schools, youth centers, and therapists willing to participate, providing insights into broader program acceptance and feasibility.

### Implementation

We will examine the effectiveness of data collection and participant engagement on Instagram, which is critical for understanding how the program can be successfully implemented in a larger trial.

### Maintenance

A 6-month follow-up will gauge the long-term impact of the intervention on intended outcomes and evaluate interaction and retention rates. These data are crucial for understanding the sustainability of the program’s effects.

The operationalization of these dimensions is summarized in Table 2. The trial will be conducted in a single-group design.

**Table 2:** Overview of the questionnaires used with a single-group design to assess primary and secondary outcomes in preintervention, postintervention, and 6-month follow-up surveys.

Outcome and construct					Questionnaire		Reliability
**Primary outcomes**							
	**Life skills**						
		Decision-making or goal setting		Subscale “goal setting” of the Life Skills Ability Scale (LSAS) [73]		α=.89	
		Problem-solving		Subscale “problem solving” of the Life Skills Ability Scale (LSAS) [73]		α=.85	
		Creative and critical thinking		Self-generated items to test knowledge based on the developed program content		—^a^	
		Communication		Subscale “communication” of the Life Skills Ability Scale (LSAS) [73]		α=.78	
		**Interpersonal skills and empathy**					
			—	Questionnaire on resources in childhood and adolescence (FRKJ 8-16) [74]		α=.68-.89	
			—	Subscale “Social skills” of the Life Skills Ability Scale (LSAS) [73]		α=.82	
		Self-confidence and self-efficacy		Questionnaire on resources in childhood and adolescence (FRKJ 8-16) [74]		α=.68-.89	
		Dealing with emotions		Emotion regulation questionnaire [75]		α=.74-.76	
		**Dealing with stress**					α=.69-.88
			—	Stress- and Coping-Inventory (SCI)		—	
			—	Subscales “Physical symptoms” + “Coping strategies” [76]		—	
	**Social media–related skills**						
		Cyberbullying		European Cyberbullying Intervention Project Questionnaire (ECIPQ) [77]		α=.96	
		**Media-based empathy**					α=.80
			—	Media-Based Empathy (MBE)		—	
			—	Subscale “Cognitive Media Empathy with Real Persons” [78]		—	
		**Social media use**					
			—	Social media use time		—	
			—	Social media disorder scale-short form [79]		α=.81	
			—	Bergen social media addiction scale [80]		*r_tt_*=.82	
		Smartphone use		Smartphone addiction scale [81]		α=.85	
**Secondary outcomes**							
	**Risk behavior**						
		**Substance use**					
			—	Alcohol Use Disorder Identification Test (AUDIT) [82]		α=.80	
			—	Cigarettes and cannabis - questions from the HBSC study [83]		—	
	**Health status**						
		Subjective state of health		Subjective state of health [84,85]		—	
		Well-being		World Health Organization-5 well-being-index [86]		α=.92	
		**Life satisfaction**					
			—	German version of the Satisfaction with Life Scale (SWLS) [87,88]		α=.87	
			—	Short scale for the assessment of general life satisfaction (L-1) [89]		*r_tt_*=.67	
		Mental health: anxiety and depression		Hopkins Symptom Checklist-25 (HSCL-25) [90]		α=.94	
		School performance		School grades (German, English, and Math), grade point average, and change		—	
**Covariants**							
	**General**						
		**Acceptance and feasibility of the program**					—
			—	Self-generated items based on the Training Evaluation Inventory (TEI) [91]		—	
			—	Net-Promoter-Score		—	
		**Sociodemographic variables**					
			—	Gender, age, migration background, socioeconomic status: family affluence scale [92,93]		*r_tt_*=.90	
			—	Subjective Socioeconomic Status scale (SSS-Scale) [94]		α=.70	

^a^Not applicable.

### Recruitment

This accessibility study will recruit participants via schools, youth centers, and therapeutic facilities across Germany. Adolescents from various school types (grammar, secondary, comprehensive, and independent) and socioeconomic backgrounds are targeted. Recruitment spans several federal states (Berlin, Mecklenburg-Western Pomerania, North Rhine-Westphalia, Saxony, Lower Saxony, and Thuringia), chosen for representativeness and synchronized holidays, encompassing both rural and urban areas. State school participation requires ethical approval and ministry or school board consent. Schools, youth centers, and therapeutic facilities are randomly selected within these states.

Recruitment will begin once approvals are obtained, with invitations issued in waves based on response rates. Teachers, youth workers, and therapists initially agreed to participate (Multimedia Appendix 1). Adolescents are then recruited through digital information events, which aim to build motivation to participate through highlighting the relevance of the programs’ content for their life stage (Multimedia Appendix 2). In addition to research findings on that, key adolescent concerns were identified in the pilot study. The use of Instagram minimizes participation effort and the recruitment in schools fosters group dynamics. Further, the program incentivizes engagement with rewards and ensures anonymity and confidentiality, creating a safe space for discussing sensible and at the same time interesting topics. Finally, together with their parents, adolescents agree to participate in the study.

To join the study, participants need an Instagram account, must be proficient in German, and must be aged 14-17 years. The recruitment goal includes 2 classes from grammar schools, 3 classes from secondary schools, and 1 class from an independent school per state, plus 20 adolescents from youth centers and therapeutic institutions in each state, accommodating additional participants if interested.

As we are taking an innovative approach, it is not clear how complete the recruitment will be. The sample size depends on adolescents who voluntarily want to participate. Since adolescents work on the program in their free time, it can be assumed that fewer will participate than in compulsory programs conducted in school. Recruitment will start in May 2023 and end as soon as the target criteria are met or the program starts in September.

### Data Collection

**Figure 1:**
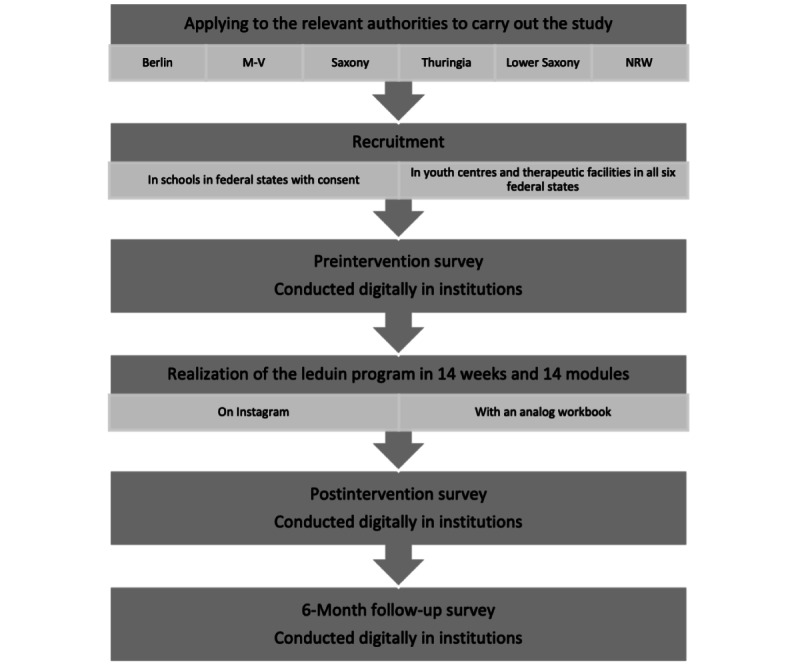
Flowchart illustrating recruitment process, single-group design (1 intervention group), and points of assessment (preintervention, postintervention, and 6-month follow-up surveys). M-V: Mecklenburg-Western Pomerania; NRW: North Rhine-Westphalia.

In this study, both qualitative and quantitative data are gathered to explore the RE-AIM framework’s dimensions, involving (1) documentation by the recruitment team and (2) personal-level outcomes via questionnaires. Figure 1 illustrates the study design with the recruitment phase and points of assessment.

To assess RE-AIM’s aspects of reach, acceptance, and implementation, data will be collected on the number of schools, youth centers, and therapeutic facilities contacted per state; their response rates; information events held; and consents after the event. The recruitment team will also document institutions’ responses.

Personal level characteristics outcomes, measuring reach, effectiveness, and maintenance, are gathered through web-based questionnaires in a preintervention, postintervention, and follow-up design. Participants and institutions receive unique codes for tracking. The institutional link aims for support through teachers or youth workers and therapists and thus comprehensive data collection by conducting the entire study, including data collection, within 1 school year. Schools optionally receive supplementary teaching materials for the leduin program content.

The following constructs are collected by means of questionnaires: primary outcomes (life skills [decision-making or goal setting, problem-solving, creative and critical thinking, communication, interpersonal skills and empathy, self-confidence and self-efficacy, managing emotions, and managing stress], and social media–related skills [media-based empathy, cyberbullying, smartphone, and addictive or disordered social media use]); secondary outcomes (health status [well-being, subjective health and mental health, and life satisfaction], risk behavior [substance use], and school performance); and covariants (acceptance and feasibility of the program and sociodemographic variables). Table 2 lists the survey instruments used to measure the constructs just described, including reliability.

For the web-based data collection of the participants’ personal data, we use the SoSci Survey platform with which we have an order data processing agreement for the leduin study that has been reviewed by the data protection officer of the University of Greifswald. This ensures the technical and organizational measures required for data security. The provider SoSci Survey works with SSL encryption (HTTPS) of the data when filling out the questionnaire and when retrieving the collected data. A secure SSL configuration (Qualys SSL Labs) secures the transmission of the data. The servers of the University of Greifswald are used to store the data. The data are secured by daily backup and are usually stored for 90 days (3 months).

The initial data collection in the pilot study proved successful due to teacher involvement. However, transferring responsibility for the subsequent data collection to the adolescents led to significant gaps in the data. To address this in the accessibility study, we will prioritize early emphasis on the importance of data collection and ensure consistent supervision of the adolescents to mitigate missing data issues.

### Data Management

Participation in the study is voluntary, with participants receiving detailed information about the procedure and providing informed consent according to the Declaration of Helsinki. This consent can be withdrawn anytime without justification or repercussions. Pseudonymized data can be deleted until fully anonymized, a detail made clear in the study information.

The data are stored on password-protected servers at the University of Greifswald, secured by daily backups. Access is restricted to authorized scientific personnel for qualitative and quantitative analysis. Project management and data processing staff at the university ensure data plausibility, completeness, and accuracy. After collection, data are quickly anonymized.

Data management adheres to the German Psychological Society’s recommendations, focusing on quality assurance, knowledge optimization, and maximizing cost-benefit ratios. Cleaned, anonymized primary data, along with associated syntax commands, will be openly accessible in line with open science principles, a fact communicated to participants in the study information.

### Data Analysis

#### Statistical Methods

Quantitative analyses will vary according to the different dimensions of the RE-AIM framework [71]. The software R Studio (Posit) and SPSS (IBM Corp) will be used [95]. Multilevel logistic regressions will be conducted to evaluate the dimensions of reach, acceptance, and implementation. Here, the extent to which the following variables influence institutions’ initial interest in the program, individuals’ initial willingness to participate in the program, completeness of data, and individual engagement within the program is examined: socioeconomic status, gender, age, mode of recruitment (school, youth center, and therapeutic facility), school type, performance, well-being, life satisfaction, and mental health. The initial interest of institutions in the program is operationalized by the proportion of institutions (schools, youth centers, and therapeutic institutions) that participated in an information session. The initial willingness of individuals to participate in the program is assessed by the percentage of students who decide to participate in the program after an information session. The completeness of data is examined by the percentage of data in the preintervention, postintervention, and follow-up surveys, and individual engagement is measured by the participants’ self-assessment.

For quantitative analyses of the dimensions of reach, effectiveness, and sustainability, mixed-effect multilevel regression analysis with institutions (school, youth center, and therapeutic facility), classes, and individuals as evaluation levels and 3 measurement points (preintervention, postintervention, and follow-up) will be carried out. The multilevel structure will be assumed if the actual design effect resulting from the intraclass correlation is >2 [96]. The influence of covariants such as gender, age, and socioeconomic status will be controlled. In addition, the individual courses in the outcome variables, as described before, will be examined by using latent growth models across the 3 time points. Logistic regressions will be conducted to investigate the impact of the program on participants’ addictive or disordered social media use by comparing pre- and postintervention data.

Qualitative analyses of the recruitment process will be executed by conducting a qualitative content analysis [97] using the software MAXQDA (VERBI Software). This should provide information on the barriers to participation in the program and also identify the facilitators.

Table 3 summarizes the different dimensions of the RE-AIM framework, their operationalization, and corresponding statistical methods.

**Table 3:** Dimensions of the reach, effectiveness, adoption, implementation, and maintenance (RE-AIM) framework, operationalization, and statistical methods to evaluate a social media–based life skills program (leduin).

Dimension	Operationalization	Statistical method
Reach	Dependent variables Initial interest Institutional level: number of institutions (schools, youth centers, and therapeutic facilities) contacted per federal state and positive responses Individual level: proportion of adolescents who agree to participate Qualitative process information of facilitators and carriers Independent variables from questionnaires Socioeconomic status, gender, age, mode of recruitment (school, youth center, and therapeutic facility), school type, well-being, life satisfaction, and mental health	Multilevel logistic regressions Qualitative content analysis
Effectiveness	Primary outcomes: Life skills and social media–related skills Secondary outcomes: mental health, well-being, risk behavior, school performance	Mixed-effect multilevel regression analysis with institutions (school, youth center, and therapeutic facility), classes, and individuals as evaluation levels and 3 measurement points (preintervention, postintervention, and follow-up) Latent growth models Logistic regressions
Acceptance	Dependent variables Willingness to participate: Number of information events Number of consents Individual engagement Independent variables from questionnaires Socioeconomic status, gender, age, mode of recruitment (school, youth center, and therapeutic facility), school type, well-being, life satisfaction, and mental health Qualitative process information of facilitators and carriers	Multilevel logistic regressions Qualitative content analysis
Implementation	Dependent variables Completeness of data collection in the different institutions Participation of adolescents in the program on Instagram (active and passive) Independent variables from questionnaires Socioeconomic status, gender, age, mode of recruitment (school, youth center, and therapeutic facility), school type, well-being, life satisfaction, and mental health	Multilevel logistic regressions
Maintenance	Primary outcomes: life skills and social media–related skills Secondary outcomes: mental health, well-being, risk behavior, school performance Participation of adolescents in the program on Instagram (active and passive)	Mixed-effect multilevel regression analysis with institutions (school, youth center, and therapeutic facility), classes, and individuals as evaluation levels and 3 measurement points (preintervention, postintervention, follow-up) Latent growth models Logistic regressions

#### Monitoring

Since the leduin study is not a clinical study, it is not expected that the participants will experience any harm. Therefore, the use of a data monitoring committee is waived. However, communication within the program will be monitored by an internal monitoring team in accordance with netiquette [98]. Violations of netiquette will be commented on and discussed within the program, and if necessary, the authors will be excluded from the program if there is no improvement. If harm is unexpectedly caused to a participant during the program, the program will be interrupted if there are indications that the harm may have been caused by the program. After review, the program will either be revised or continued.

### Ethical Considerations

The leduin study has been positively assessed by the ethics committee of the Greifswald University Medical School (BB 190/22). Approval for the study has also been sought from the relevant education authorities and ministries for state schools in Germany. The data protection regulations have been developed with the data protection officer of the University of Greifswald and a register of processing activities is available. If important protocol modifications are made, this will be communicated to the responsible ethics committee, the school authorities and ministries, the data protection officer, and the study participants.

Participating adolescents and their parents consent to their participation in the study. Consent is given on the basis of the study information and information events.

### Dissemination Policy

Trial results will be communicated to participants, schools, prevention professionals, and authorities via publication and in edited form in a brochure and on the project website, further through publications, conference contributions at academic conferences, and practitioners’ congresses (eg, Digitallabor and Medienschule).

## Results

Recruitment for the study was planned from May 2023 until the beginning of the leduin program in October 2023. As of March 2024, a total of 283 participants agreed to participate in the study.

## Discussion

### Overview

Social media offers effective ways of reaching a broad audience and can be used to address risk behavior and skill development [3,15,22]. To this aim, life skills can be a holistic approach. Despite the prevalence of digital training, few are evidence-based and access to the target audience remains challenging [27,29,30,33]. Moreover, existing life skills training often fails to address digital challenges. Further, the potential of using existing social media to reach adolescents has not been exploited. Our program is designed to improve digital life skills using Instagram, a platform that aligns with adolescents’ habits and offers interactive features suitable for e-learning. The program addresses both the benefits and the risks associated with social media use. Our upcoming study aims to assess the accessibility of this innovative approach, leveraging existing social media to develop digital life skills, with future plans to evaluate its effectiveness.

In a pilot study, the leduin program was tested with 101 students in 9th and 10th grade across schools in North Rhine-Westphalia and Lower Saxony, Germany (E Zimmermann and S Tomczyk, unpublished data, 2024). The study, conducted from November 2022 to February 2023, involved interviews with 14- to 17-year-old participants and their teachers to evaluate the program’s acceptance and initial effectiveness. Results showed positive reception and commitment, with participants reporting beneficial learning outcomes in social media use, self-care, and stress reduction. However, our pilot study highlighted substantial challenges in reaching the target group. While some classes were very interested in participating, others showed minimal involvement. This disparity suggests that simply making the program available is not sufficient. Effective strategies are needed to actively engage adolescents, ensuring they are both aware of and interested in participating. The leduin program is crafted to subtly enhance life skills through everyday activities with minimal effort over an extended period with spaced learning, aligning with effective learning and skill acquisition theories [69,70]. While social media proves effective in health interventions [22,30], participation depends on the individual’s choice. Accordingly, adolescents might not fully appreciate the personal benefits of such engagement in their developmental stage [9]. To address this, we aim to reach adolescents through institutions already engaged in preventative work. However, since the intervention occurs during leisure time, even with its low barriers, consistent participation cannot be assured. Additionally, there is a possibility of waning intrinsic motivation over time.

The objectives of the accessibility study focus on identifying effective methods to engage adolescents in a digital life skills program on social media. It aims to encourage participation and sustain engagement to improve digital life skills. The study uses the RE-AIM model to guide its design, primarily focusing on the dimension “reach”. Accordingly, key aspects include reaching a representative adolescent sample, examining the characteristics of consenting participants, and analyzing the variance in engagement levels. We thus aim to assess the accessibility of a social media intervention amidst heightened stress levels in schools and among adolescents post–COVID-19 pandemic, as indicated by our pilot study. Despite the critical need for prevention work, overall accessibility may be significantly constrained due to resource limitations, independent of the program’s specifics.

The study further aims to identify relevant aspects of the other dimensions of the RE-AIM framework (effectiveness, acceptance, implementation, and maintenance) and thus examines the program’s effects on life skills and social media–related skills, along with health, risk behavior, and school performance. It assesses program acceptance among schools, youth centers, and therapeutic facilities; the effectiveness of data collection, participant interaction, and retention rates on Instagram; and the long-term impact of the intervention. While this approach may yield valuable information, further studies must follow to fully evaluate the aspects of the comprehensive RE-AIM model.

### Limitations

The limitations of the described approach include potential sampling bias, as engagement levels and the willingness of schools and youth centers to participate may not represent the broader adolescent population. Schools and youth centers facing significant challenges could be unable to participate in the study due to capacity constraints. This stress could be related to having a particularly burdened student body, affecting representativeness. Moreover, while directly recruiting adolescents for a social media intervention via social media platforms might initially appear to be an effective strategy, it presents substantial challenges in the context of a scientific study. The primary issue lies in the difficulty of reliably verifying adolescents’ identities and securing parental consent in an online environment. Additionally, while data collection itself is not intended to be conducted through social media, motivating adolescents to consistently participate in data collection becomes challenging without a supervised setting. This lack of direct oversight and engagement can significantly impact the reliability and consistency of data collection. Therefore, this approach is considered unfeasible for maintaining the rigorous standards required for scientific research.

The quasi-experimental design, while useful, might not fully account for confounding variables influencing engagement and outcomes. We are launching the leduin program with a digital information event, aiming for cost-effectiveness and wide reach among adolescents from diverse regions. This event serves to arouse interest. However, it is essential to thoroughly explain the scientific study’s framework accompanying the program for informed consent. While this detailed explanation may dampen interest, it is a necessary step to ensure ethical compliance and informed participation. Consequently, we will only be able to examine the characteristics of adolescents who initially agreed to participate in the study and take part in the follow-up survey. This will limit representativeness. Since we are also dependent on the voluntary participation of the participants, dropouts are to be expected, both in the program itself and in the surveys.

Additionally, relying on social media platforms, Instagram could limit reach to adolescents not actively using or engaging with this platform. The follow-up period of 6 months, although significant, may not be sufficient to fully assess the long-term sustainability of the intervention’s effects. Further, our sample size for evaluating the effectiveness of the intervention is calibrated for detecting medium-sized effects, which, while possible, are not guaranteed.

### Conclusions

Our aim is to deepen our understanding in this field by identifying effective access strategies for social media interventions. Accessibility is crucial, acting as the “eye of the needle” for such interventions. Even the most effective programs cannot achieve their intended impact if they fail to reach and be accepted by the target group. By successfully navigating this pivotal aspect, we can ensure that our interventions are both accessible and effective, maximizing their potential benefits. We recognize the dual role of social media in interventions. On the one hand, these platforms offer valuable tools for reaching and engaging our target audience. On the other, it is imperative to acknowledge and address the inherent risks associated with social media use. Neglecting these risks could lead to unintended negative consequences, following a “dark-logic-model” where interventions might inadvertently cause harm. Thus, our focus is not only on leveraging social media for positive outcomes but also on mitigating its potential adverse effects to ensure a safe and effective intervention environment.

## Data Availability

Data sharing is not applicable to this article as no data sets were generated or analyzed during this study.

## Appendix

Multimedia Appendix 1 Study information and declaration of consent for the leduin study for caregivers.

Multimedia Appendix 2 Study information and declaration of consent for the leduin study for adolescents.

## Abbreviations

RE-AIM: reach, effectiveness, adoption, implementation, and maintenance
