# Supplementary material for: Fostering Digital Life Skills Through Social Media With Adolescents in 6 German States: Protocol for an Accessibility Study According to the RE-AIM Framework
Source: JMIR Res Protoc. 2024 Apr 17;13:e51085. doi: 10.2196/51085 (PMC11063895; doi:10.2196/51085)
Supplement: Multimedia Appendix 1 [file resprot_v13i1e51085_app1.pdf]

12.3 Studieninformationen Lehrkräfte

Studieninformation zur Teilnahme am Programm „*leduin*“ und zur Verwendung personenbezogener Daten aus Fragebögen für die Studie „*leduin* – Lebenskompetent durch Instagram“ für Lehrkräfte/Jugendhilfe/Therapeut*innen

Sehr geehrte Lehrkraft/Jugendhilfe/Therapeut*in,

unser Forschungsteam von der Universität Greifswald hat das Programm „*leduin* – Lebenskompetent durch Instagram“ entwickelt, welches die Lebenskompetenzen von Jugendlichen der 10. Klasse mittels Instagram fördern soll. Wir möchten Sie daher fragen, ob Sie mit Ihrer Klasse/den Jugendlichen in Ihrem Jugendclub/den Jugebdlichen auf Ihrer Warteliste an dem *leduin*-Programm und der begleitenden Studie teilnehmen möchten/ihnen das Programm vermitteln wollen. Ob Ihre Klasse/Ihre Gruppe/die Jugendlichen auf Ihrer Warteliste teilnimmt/teilnahmen, entscheiden zunächst Sie. Im Anschluss sollen die Jugendlichen und Eltern um die Einwilligung in die Studienteilnahme gebeten werden. Toll wäre es, wenn Sie Ihre Klasse/Ihre Gruppe als Kollektiv zur Teilnahme ermutigen könnten. Auf diese Weise wollen wir sowohl hoch motivierte als auch lern- oder motivationsschwächere Jugendliche erreichen.

Warum wollen wir Lebenskompetenzen fördern?

Lebenskompetenzen unterstützen Menschen dabei, die vielfältigen Herausforderungen des täglichen Lebens zu meistern. Diese können auch in den sozialen Medien auftreten: Mobbing, Fake News, Datenklau, unrealistische Erwartungen… Herausforderungen gibt es heutzutage online wie offline. Und genauso gibt es Kompetenzen, die uns Menschen dabei helfen: Lebenskompetenzen – kurz Life Skills. Die Förderung von Lebenskompetenzen bei Jugendlichen hat somit weitreichende positive Auswirkungen, wie etwa verbesserte körperliche und psychische Gesundheit, erhöhtes Wohlbefinden, bessere schulische Leistungen, ein höheres Selbstwertgefühl oder die Reduzierung von Risikoverhalten wie Alkohol- und Drogenkonsum. Genau darauf soll das *leduin*-Programm über die Förderung von Life Skills einwirken.

Was ist das Programm „*leduin* - Lebenskompetent durch Instagram“?

„*leduin*“ ist ein modernes Programm zur Lebenskompetenzförderung für das Smartphone. Bisherige Lebenskompetenz-Programme werden meist während des Unterrichts durchgeführt, wozu in vielen Schulen die Zeit fehlt. Auch ist es für Jugendliche oft unangenehm, vor der Lehrkraft oder der Klasse/der Gruppe über ihre privaten Wünsche, Gefühle oder Probleme zu reden. Wir haben an der Universität Greifswald ein Programm entwickelt, das über soziale Medien läuft. Jugendliche nutzen soziale Medien durchschnittlich 3 Stunden am Tag. Innerhalb dieser Zeit sollen sie auch die Inhalte des *leduin*-Programms durchlaufen. Wir nutzen somit die große Beliebtheit und Nutzungsdauer von sozialen Medien und fördern Lebenskompetenzen niedrigschwellig, stetig und im Alltag der Jugendlichen. Gleichzeitig sollen die Jugendlichen durch Lebenskompetenzen auch für eine verantwortungsvolle und funktionale Social Media-Nutzung sensibilisiert und befähigt werden.

Was passiert bei der Studie?

Wir haben das *leduin*-Programm zusammen mit Jugendlichen, Lehrkräften, Expertinnen und Experten sowie auf der Grundlage psychologischer Forschung erarbeitet. In einer Pilotstudie haben wir bereits getestet, dass das Programm sehr gut funktioniert. Die teilnehmenden Jugendlichen haben davon sehr profitiert. Jetzt wollen wir in einer größeren Studie die Erreichbarkeit des Programms untersuchen – wie Jugendliche am besten von der Teilnahme überzeugt werden können, welche Effekte das Programm auf individueller Ebene hat, wie es akzeptiert und nachhaltig umgesetzt wird.

Zunächst wollen wir Sie also bitten, das *leduin*-Programm Ihren Schülerinnen und Schülern/betreuten Jugendlichen/Jugendlichen auf der Warteliste vorzustellen – hierfür bieten wir eine digitale Infoveranstaltung an, in der wir das Programm in ca. 15 Minuten vorstellen. Sie erhalten Infomaterial für Jugendliche und Eltern. Vorher erhalten Sie selbstverständlich die Möglichkeit, alle Ihre Fragen zum *leduin*-Programm zu stellen.

Die Jugendlichen, die an dem Programm teilnehmen, bitten wir dann, einmal vor und einmal nach dem Programm und 6 Monate später einen Fragebogen auszufüllen. Auf diese Weise können wir feststellen, ob es eine Entwicklung gegeben hat. Die ersten Fragebögen sollen Ende August/Anfang September 2023 in den ersten Wochen nach den Sommerferien möglichst digital ausgefüllt werden.

Die Vermittlung der Programminhalte erfolgt dann in der Freizeit der Jugendlichen von September bis Dezember 2023 für 14 Wochen bis zu den Weihnachtsferien. Es wird täglich Beiträge auf Instagram in Form von Videos, Texten, Umfragen, Quizzen, Anregungen und Challenges geben. Die Jugendlichen können die Beiträge anschauen, wann immer sie das möchten – wahrscheinlich in der Zeit, in der sie Instagram sowieso nutzen. Das Projektteam steht Ihnen selbstverständlich während der gesamten Zeit für Rückfragen zur Verfügung. Optional erhalten Sie Unterrichtsmaterialien zu den drei Themenkomplexen Individual-, Sozial- und Gesundheitskompetenzen, um die im *leduin*-Programm behandelten Inhalte bei Bedarf oder Interesse zu wiederholen oder zu vertiefen.

Welche Risiken sind für die Schülerinnen und Schüler/Jugendlichen mit der Teilnahme verbunden?

Wir haben bei der Entwicklung des *leduin*-Programms sehr großen Wert darauf gelegt, die Jugendlichen zu schützen. So wird das Programm über einen privaten Account vermittelt, wodurch nur berechtigte Jugendliche an dem Programm teilnehmen können und der Austausch in einem geschützten und nachvollziehbaren Rahmen stattfindet. Uns ist bewusst, dass die Nutzung sozialer Medien prinzipiell mit Herausforderungen einhergeht. Ein Teil des *leduin*-Programms ist es daher, genau für diese Herausforderungen Kompetenzen zu vermitteln: wie die Jugendlichen mit belastenden Inhalten umgehen können. Und wie sie den eigenen Social Media-Konsum so gestalten können, dass es ihnen dabei gut geht. Innerhalb des Programms gibt es außerdem Vereinbarungen, wie die Teilnehmenden auf wertschätzende Art miteinander kommunizieren. Gleichzeitig sprechen wir über Probleme, Sorgen und Gefühle. Das kann auch mal belastend sein. Die Studienleitung ist während des gesamten Programms auf Instagram präsent und eine direkte Ansprechperson. Auch wissen wir, dass das Ausfüllen der Fragebögen von

vielen als langweilig empfunden wird. Wir haben uns deshalb bemüht, den Fragebogen so kurz wie möglich zu gestalten.

Bestimmungen zum Datenschutz

Sofern Sie sich bereit erklären, mit Ihrer Klasse/Ihrer Gruppe an dem *leduin*-Programm teilzunehmen, verarbeiten wir Ihren Namen, die Schule/Institution, an der Sie tätig sind, sowie die Klasse/Gruppe, die Sie betreuen. Zudem benötigen wir Ihre Mailadresse, um Ihnen Information zu dem Programm und der Studie zukommen zu lassen und zwecks organisatorischer und weiterer begleitender Aspekte mit Ihnen in Kontakt zu bleiben. Nur berechtigte Mitglieder des Forschungsteams haben Zugriff auf die Daten. Eine Weiterleitung an andere Empfänger erfolgt nicht. Die Teilnahme Ihrer Klasse/Gruppe an dem Programm sowie an dem Interview erfolgt ausschließlich auf Grundlage Ihrer Einwilligung gem. Art. 6 Abs. 1 lit. a DSGVO.

Sie haben das Recht, Ihre Einwilligung in die Datenverarbeitung jederzeit und ohne Angabe von Gründen schriftlich zu widerrufen. Ab diesem Zeitpunkt erheben oder verwenden wir Ihre Daten dann nicht mehr. Hierfür können Sie sich an das Forschungsteam wenden (Elizabeth Zimmermann: elizabeth.zimmermann@uni-greifswald.de).

Darüber hinaus haben Sie das Recht, auf Antrag unentgeltliche Auskunft über Ihre gespeicherten personenbezogenen Daten zu bekommen. Das gilt für deren Herkunft und Empfänger, den Zweck und die Dauer der Datenverarbeitung. Zusätzlich haben sie unter bestimmten Voraussetzungen das Recht auf Berichtigung, auf Einschränkung der Verarbeitung (z. B. Sperrung), auf Löschung, auf die Datenübertragbarkeit Ihrer Daten sowie auf Widerspruch gegen Direktwerbung.

Wenn Sie finden, dass wir beim Datenschutz Fehler gemacht haben, können Sie sich beschweren. Wenden Sie sich dafür schriftlich an die zuständige Aufsichtsbehörde. Sie wird Ihre Beschwerde prüfen:

Der Landesbeauftragte für Datenschutz und Informationsfreiheit Mecklenburg-Vorpommern
Werderstraße 74a, 19055 Schwerin
E-Mail: info@datenschutz-mv.de

Datenschutzbeauftragter der Universität Greifswald
Herr René Schülke
SIS - Schweriner IT- und Servicegesellschaft mbH
Eckdrift 93, 19061 Schwerin
E-Mail: datenschuetzer@uni-greifswald.de

Rechtliche Grundlagen

Die Universität Greifswald arbeitet nach den Vorschriften der Datenschutz-Grundverordnung, des Bundesdatenschutzgesetzes, des Datenschutzgesetzes M-V und allen anderen datenschutzrechtlichen Bestimmungen. Datenschutzrechtlich verantwortlich ist die

Universität Greifswald
Gesetzlich vertreten durch die Rektorin Prof. Dr. Katharina Riedel
Domstraße 11, 17489 Greifswald
Telefon: 03834 420 0
E-Mail: rektorin@uni-greifswald.de

Veröffentlichung der Forschungsergebnisse

Die Veröffentlichung von Forschungsergebnissen in Publikationen oder auf Tagungen erfolgt ausschließlich in vollständig anonymisierter Form. Nach Abschluss dieser Studie übergeben wir die anonymisierten Daten an ein professionelles Forschungsdatenzentrum. Dort werden die Daten sicher aufbewahrt. In dem Datenzentrum stehen die Daten anderen Forschenden zu wissenschaftlichen Zwecken zur Verfügung. Sie werden stets vertraulich unter Wahrung der Datenschutzgesetze behandelt. Alle anonymisierten Daten werden nach 10 Jahren gelöscht.

Haben Sie weitere Fragen?

Sollten Sie noch Fragen zu der Studie haben, können Sie sich mit Ihren Fragen an die Projektleitung wenden:

Projektleitung Elizabeth Zimmermann
Instagram: leduin_official
Telefon: 03834 420 3807
E-Mail: leduin@uni-greifswald.de

Wir würden uns sehr freuen, Sie und Ihre Klasse/Gruppe für die Teilnahme an unserer Studie gewinnen zu können!

Mit freundlichen Grüßen

Jun.-Prof. Dr. Samuel Tomczyk M. Sc. Elizabeth Zimmermann

12.3.1 Einwilligungserklärung Lehrkräfte

Einwilligungserklärung zur Teilnahme am Programm „*leduin*“ und zur Verwendung personenbezogener Daten aus Fragebögen für die Studie „*leduin* – Lebenskompetent durch Instagram“ für Lehrkräfte/Jugendhilfe/Therapeut*innen

Voraussetzung für die Teilnahme Ihrer Klasse an dem *leduin*-Programm ist, dass Sie bereit sind, diese Studie zu begleiten. Wenn ja, bitten wir sie, die entsprechenden Häkchen zu setzen und die Einwilligungserklärung abzuschicken.

☐ Ich habe die Studieninformation gelesen und verstanden. Alle meine Fragen wurden zu meiner Zufriedenheit beantwortet und ich hatte genügend Zeit, die Teilnahme zu bedenken. Ich willige in die Teilnahme meiner Klasse/Gruppe an der *leduin*-Studie ein. Hierfür werde ich das Projekt vorstellen und die Einwilligungen der entsprechenden Jugendlichen an die *leduin*-Projektleitung übermitteln.

☐ In die Erhebung und Verwendung der Daten wie in der Studieninformation beschrieben willige ich ein.

☐ Ich willige ein, dass mich das *leduin*-Team per Mail zum Zwecke der Information und der Organisation des Programms und der Studie sowie weiterer begleitender Aspekte kontaktiert.

Schule/Institution: ____

Klasse/Gruppe: ____

Mailadresse: ____

____

Name der Lehrkraft/Jugendhilfefachkraft/Therapeut*in

Vielen Dank für die Einwilligung in die Teilnahme an der Studie „*leduin* - Lebenskompetent durch Instagram“!
