# Supplementary material for: Fostering Digital Life Skills Through Social Media With Adolescents in 6 German States: Protocol for an Accessibility Study According to the RE-AIM Framework
Source: JMIR Res Protoc. 2024 Apr 17;13:e51085. doi: 10.2196/51085 (PMC11063895; doi:10.2196/51085)
Supplement: Multimedia Appendix 2 [file resprot_v13i1e51085_app2.pdf]

12.4 Studieninformationen Jugendliche und Erziehungsberechtigte

Studieninformation zur Teilnahme am Programm „leduin“ und zur Verwendung personenbezogener Daten aus Fragebögen für die Studie „leduin – Lebenskompetent durch Instagram“

Liebe Jugendliche oder lieber Jugendlicher,

wir als Forschungsteam von der Universität Greifswald haben das Programm „*leduin* – Lebenskompetent durch Instagram“ entwickelt, das die Lebenskompetenzen von Jugendlichen der 10. Klasse auf Instagram fördern soll. Wir möchten Dich fragen, ob Du am *leduin*-Programm und der begleitenden Studie teilnehmen möchtest. Das kannst Du mit Deinen Eltern zusammen selbst entscheiden. Die folgenden Informationen sollen Dir bei Deiner Entscheidung helfen.

Was sind Lebenskompetenzen und wofür brauchen wir sie?

Lebenskompetenzen sind die Fähigkeiten, die Du für die vielen Herausforderungen in Deinem Alltag brauchst. Wir sind alle mal traurig, haben Stress, Streit und Probleme. Manchmal fühlen wir uns schlecht oder einsam, haben Sorgen, sind überfordert oder mit uns und der Welt unzufrieden. Auch in den sozialen Medien können wir mit Problemen konfrontiert sein: Mobbing, Fake News, Datenklau, unrealistische Erwartungen… Herausforderungen gibt es online wie offline. Und genauso gibt es Kompetenzen, die Dir dabei helfen: Lebenskompetenzen – kurz Life Skills. Du kannst lernen, wie Du gesund mit Stress und Gefühlen umgehen kannst. Wie Du Dir Ziele setzen und diese erreichen kannst. Wie Du gute Beziehungen aufbauen und aufrechterhalten kannst. Wie Du Dich im Angesicht von Risiken kompetent verhalten kannst. So geht es auch um Süchte und Verhaltenssüchte, um Fake News und Hate Speech, sexuelle Belästigung, Cyber-Mobbing und Informationen im Internet allgemein. Du kannst Dich mit anderen Jugendlichen anonym austauschen und erhältst so Einblicke, wie es anderen in Deinem Alter geht, was sie beschäftigt und auch, was sie so für Lösungen finden.

Was ist „*leduin*“ für ein Programm?

Das *leduin*-Programm ist ein digitales Programm zur Lebenskompetenzförderung. Bisherige Lebenskompetenz-programme werden meist während des Unterrichts durchgeführt, wozu in vielen Schulen die Zeit fehlt. Auch ist es für Jugendliche oft unangenehm, vor der Lehrkraft oder der Klasse über Wünsche, Gefühle oder Probleme zu reden. Wir haben an der Universität Greifswald ein Programm entwickelt, das über Instagram läuft. Dadurch soll es gut in Deinen Alltag passen und Spaß machen. So lernst Du quasi nebenbei, Dein Leben selbst in die Hand zu nehmen.

Was passiert bei so einer Studie?

Wir haben das *leduin*-Programm zusammen mit Jugendlichen, Lehrkräften, Expertinnen und Experten und auf Grundlage psychologischer Forschung erarbeitet und auch schon einmal mit einer Gruppe von etwa 100 Jugendlichen getestet. Das war sehr erfolgreich und die Jugendlichen haben persönlich sehr von der Teilnahme profitiert. Jetzt wollen wir das Programm mit einer größeren Gruppe Jugendlicher durchführen. Auf diese Weise

können wir die beabsichtigten Effekte untersuchen. Dazu führen wir diese Studie durch. Deshalb bitten wir Dich, einmal vor und einmal nach dem Programm und 6 Monate später einen Fragebogen auszufüllen. Auf diese Weise können wir uns angucken, ob das Programm auch bei vielen Jugendlichen Auswirkungen hat. Das Programm läuft für 14 Wochen von September bis Dezember 2023. Du schaust Dir täglich die Beiträge an und zwar, wann immer Du möchtest. Der Aufwand für Dich ist also minimal. Gleichzeitig lernst Du etwas, was Dir wirklich etwas für Deine psychische Gesundheit, Dein Leben, Deine Ziele, Deine Beziehungen und Deine Zukunft bringt.

Gibt es Nachteile bei der Studienteilnahme?

Wir haben bei der Entwicklung des *leduin*-Programms sehr großen Wert darauf gelegt, Dich und die anderen Jugendlichen zu schützen. Das Programm wird über einen privaten Account vermittelt, wodurch nur berechtigte Jugendliche teilnehmen können und der Austausch in einem geschützten und nachvollziehbaren Rahmen stattfindet. Uns ist bewusst, dass die Nutzung sozialer Medien prinzipiell mit Herausforderungen einhergeht. Im *leduin*-Programms vermitteln wird Dir daher genau für diese Herausforderungen Kompetenzen, so dass Du Deinen Social Media-Konsum so gestalten kannst, dass er Dir gut tut. Innerhalb des Programms gibt es außerdem Vereinbarungen, wie wir respektvoll miteinander kommunizieren. Gleichzeitig sprechen wir über Probleme, Sorgen und Gefühle. Das kann auch mal belastend sein. Wir sind während des gesamten Programms auf Instagram für Dich da und Du kannst Dich auch direkt an uns wenden. Auch wissen wir, dass das Ausfüllen der Fragebögen von vielen als langweilig empfunden wird. Wir haben uns deshalb bemüht, den Fragebogen so kurz wie möglich zu gestalten.

Freiwilligkeit der Teilnahme

Deine Teilnahme an der Studie ist freiwillig. Du kannst ablehnen und jederzeit, auch ohne Angabe von Gründen, Deine Teilnahme an der Studie beenden. Dadurch entstehen Dir keinerlei Nachteile.

Bestimmungen zum Datenschutz

Um den Erfolg des Programms untersuchen zu können, erheben wir von Dir Daten zu Forschungszwecken. Dies sind soziodemographische Daten, allgemeine Personendaten und Gesundheitsdaten auf den Rechtsgrundlagen Art. 6 Abs. 1 lit. a DSGVO sowie Art. 9 Abs. 2 lit. a DSGVO (Einwilligung). Wenn Du die Fragebögen zur Studie ausfüllst, erstellst Du einen persönlichen Code. So können wir Deine Fragebögen vor und nach dem Programm einander zuordnen. Wir speichern dabei jedoch nicht Deinen Namen und können Deine Aussagen Dir als Person nicht zuordnen. Das nennt man Pseudonymisierung, eine übliche Vorgehensweise in der Wissenschaft. Nur berechtigte Mitglieder des Forschungsteams haben Zugriff auf die Daten. Eine Weiterleitung an andere Empfänger erfolgt nicht.

Auch Deine Reaktionen auf die Instagram-Beiträge innerhalb des Programms werden auf diese Weise pseudonymisiert gespeichert. Wir nutzen für unsere Studie Daten aus den Fragebögen und die Daten von Instagram. Dies umfasst Kommentare, Likes, Eingaben in Fragensticker, Ergebnisse aus Umfragen und Quizzen und sonstige generierte Inhalte innerhalb des Programms. Weitere Daten, die durch die Instagram-Nutzung entstehen, nutzen wird nicht.

Die pseudonymisierten Daten werden nach Abschluss der Befragungen anonymisiert, indem wir den von Dir erstellten Code löschen. Ab diesem Zeitpunkt ist es uns dann auch nicht mehr möglich, Deine Daten zu löschen, da sie Dir nicht mehr zugeordnet werden können. Die Daten werden dahingehend ausgewertet, welche Lebenskompetenzen sich auf Instagram fördern lassen, wie das Programm bewertet wird und wie gut es funktioniert. Wir untersuchen außerdem, welche Funktionen von Instagram Euch am besten gefallen.

Innerhalb des Programms verlosen wir wöchentlich Preise in Form von Gutscheinen (Amazon, Netflix, Kino oder eine Spende). Wenn Du am Gewinnspiel teilnimmst und gewinnst, kontaktieren wir Dich per direct message über Instagram. Dann kannst Du entscheiden, ob Du uns – ausschließlich für die Übermittlung des Preises – Deine Mailadresse zur Verfügung stellen willst.

Du hast das Recht, Deine Einwilligung in die Datenverarbeitung jederzeit und ohne Angabe von Gründen schriftlich zu widerrufen. Ab diesem Zeitpunkt erheben oder verwenden wir Deine Daten dann nicht mehr. Hierfür kannst Du Dich an das Forschungsteam wenden (Elizabeth Zimmermann: elizabeth.zimmermann@uni-greifswald.de).

Darüber hinaus hast Du das Recht, auf Antrag unentgeltliche Auskunft über Deine gespeicherten personenbezogenen Daten zu bekommen. Das gilt für deren Herkunft und Empfänger, den Zweck und die Dauer der Datenverarbeitung. Zusätzlich hast Du unter bestimmten Voraussetzungen das Recht auf Berichtigung, auf Einschränkung der Verarbeitung (z. B. Sperrung), auf Löschung, auf die Datenübertragbarkeit Deiner Daten sowie auf Widerspruch gegen Direktwerbung.

Wenn Du findest, dass wir beim Datenschutz Fehler gemacht haben, kannst Du Dich beschweren. Wende Dich dafür schriftlich an die zuständige Aufsichtsbehörde. Sie wird Deine Beschwerde prüfen:

Der Landesbeauftragte für Datenschutz und Informationsfreiheit Mecklenburg-Vorpommern
Werderstraße 74a, 19055 Schwerin
E-Mail: info@datenschutz-mv.de

Datenschutzbeauftragter der Universität Greifswald
Herr René Schülke
SIS - Schweriner IT- und Servicegesellschaft mbH
Eckdrift 93, 19061 Schwerin
E-Mail: datenschuetzer@uni-greifswald.de

Rechtliche Grundlagen

Die Universität Greifswald arbeitet nach den Vorschriften der Datenschutz-Grundverordnung, des Bundesdatenschutzgesetzes, des Datenschutzgesetzes M-V und allen anderen datenschutzrechtlichen Bestimmungen. Datenschutzrechtlich verantwortlich ist

Universität Greifswald
Gesetzlich vertreten durch die Rektorin Prof. Dr. Katharina Riedel
Domstraße 11, 17489 Greifswald

Telefon: 03834 420 0

E-Mail: rektorin@uni-greifswald.de

Alle Daten, die während Deiner Teilnahme am Programm auf Instagram entstehen, unterliegen weiter auch den Nutzungs- und Datenschutzbestimmungen von Instagram. Diesen muss bei der Installation der App zugestimmt werden. Es ist Deine Entscheidung, ob Du dies tun und Instagram nutzen möchtest oder nicht. Die Verarbeitung der personenbezogenen Daten durch die Nutzung erfolgt durch Instagram selbst. Die Universität Greifswald hat darauf keinen Einfluss. Wenn Du Deine Betroffenenrechte zum Datenschutz bezüglich Instagram geltend machen möchtest, wende Dich bitte direkt an Instagram. Die Datenschutzerklärung von Instagram findest Du noch einmal hier verlinkt und hier die Nutzungsbedingungen. Oder aber im Internet direkt auf den Seiten von Instagram.

Veröffentlichung der Forschungsergebnisse

Die Veröffentlichung von Forschungsergebnissen in Publikationen oder auf Tagungen erfolgt ausschließlich in vollständig anonymisierter Form. Nach Abschluss dieser Studie übergeben wir die anonymisierten Daten an ein professionelles Forschungsdatenzentrum. Dort werden die Daten sicher aufbewahrt. In dem Datenzentrum stehen die Daten anderen Forschenden zu wissenschaftlichen Zwecken zur Verfügung. Sie werden stets vertraulich unter Wahrung der Datenschutzgesetze behandelt. Alle anonymisierten Daten werden nach 10 Jahren gelöscht.

Noch Fragen?

Solltest Du noch Fragen zu der Studie haben, kannst Du uns diese geren stellen. Auch im Nachhinein kannst Du Dich mit Deinen Fragen an uns wenden.

Projektleitung: Elizabeth Zimmermann
Lehrstuhl Gesundheit und Prävention
Universität Greifswald
Robert-Blum-Str. 13, 17489 Greifswald
Instagram: leduin_official
Telefon: 03834 420 3807
E-Mail: leduin@uni-greifswald.de

Wir würden uns sehr freuen, Dich für die Teilnahme an unserer Studie gewinnen zu können!

Mit freundlichen Grüßen

Jun.-Prof. Dr. Samuel Tomczyk

M.Sc. Elizabeth Zimmermann

12.4.1 Einwilligungserklärung Jugendliche und Erziehungsberechtigte

Einwilligungserklärung zur Teilnahme am Programm „*leduin*“ und zur Verwendung personenbezogener Daten aus Fragebögen für die Studie „*leduin* – Lebenskompetent durch Instagram“

Voraussetzung für Deine Teilnahme ist, dass Du einwilligst. Wenn ja, bitten wir Dich, die Einwilligungserklärung gemeinsam mit Deinen Eltern auszufüllen und zu unterschreiben.

☐ Ich habe die Studieninformation gelesen und verstanden. Alle meine Fragen wurden zu meiner Zufriedenheit beantwortet und wir hatten genügend Zeit, die Teilnahme zu bedenken. Ich bestätige, dass ich mindestens 14 Jahre alt bin. Ich willige in die Teilnahme an der oben genannten Studie und in die Verarbeitung meiner personenbezogenen Daten ein.

☐ Auch ich als erziehungsberechtigte Person willige in die Teilnahme meines Kindes an der *leduin*-Studie sowie in die Verarbeitung der personenbezogenen Daten meines Kindes ein. Ich habe die Studieninformation gelesen und verstanden. Alle meine Fragen wurden zu meiner Zufriedenheit beantwortet und wir hatten genügend Zeit, die Teilnahme zu bedenken.

Schule: ____

Klasse: ____

____

Name Jugendliche/Jugendlicher | Unterschrift Jugendliche/Jugendlicher

____

Name des Instagram-Accounts des*der Jugendlichen *(Das Programm wird auf einem privaten Account durchgeführt, damit nur berechtigte Jugendliche teilnehmen können und der Austausch in einem geschützten Rahmen stattfindet. Damit Du den Account abonnieren kannst, benötigen wir daher Deinen Instagram-Accountnamen.)*

____

Name erziehungsberechtigte Person | Unterschrift erziehungsberechtigte Person

Vielen Dank für die Einwilligung in die Teilnahme an der Studie „leduin - Lebenskompetent durch Instagram“!
